# Taxonomy and Phylogeny of Two New *Zosterodasys* Species (Protista: Ciliophora: Phyllopharyngea) from the Yangtze Estuary, China †

**DOI:** 10.3390/ani16121930

**Published:** 2026-06-22

**Authors:** Junqi Guo, Jiamei Jiang, Hongbo Pan

**Affiliations:** 1 Shanghai Universities Key Laboratory of Marine Animal Taxonomy and Evolution, Shanghai Ocean University, Shanghai 201306, China; junqi_g@foxmail.com (J.G.); jm-jiang@shou.edu.cn (J.J.); 2 Centre for Research on Environmental Ecology and Fish Nutrient (CREEFN) of the Ministry of Agriculture and Rural Affairs, Shanghai Ocean University, Shanghai 201306, China

**Keywords:** ciliate, new species, Synhymeniida, SSU rDNA

## Abstract

The genus *Zosterodasys* is a widely distributed group of typical algivorous ciliates. It remains poorly investigated and its phylogeny has not been well resolved yet. In the present work, two new *Zosterodasys* species, *Z. paraminutus* sp. nov. and *Z. shanghaiensis* sp. nov., isolated from an intertidal zone in Shanghai, China, were investigated based on both microscopic observation of live and protargol-stained specimens and SSU rDNA analyses. Both new species possess a unique S-shaped synhymenium that completely encircles the body. *Zosterodasys paraminutus* sp. nov. is characterized by a body size of about 87–130 × 28–50 μm, an elongated oval body shape, 39–59 somatic kineties, 12–15 nematodesmal rods, and numerous contractile vacuoles arranged along both sides of the cell. *Zosterodasys shanghaiensis* sp. nov. is characterized by a 114–174 × 50–85 μm body size, an obovate body shape, 74–122 somatic kineties, 12–17 nematodesmal rods, and several contractile vacuoles scattered throughout the body. Phylogenetic analysis questions the monophyly of *Zosterodasys*, since *Zosterodasys* sp.5 and *Zosterodasys* sp.6 diverged from its congeners, indicating these two sequences might be misidentified. In addition, we also provide an updated identification key for the genus *Zosterodasys*.

## Simple Summary

Ciliates are tiny but important single-celled organisms in aquatic ecosystems. As a typical benthic ciliate group, the genus *Zosterodasys* remains poorly investigated and its phylogeny has not been well resolved yet. In this study, *Z. paraminutus* sp. nov. and *Z. shanghaiensis* sp. nov. collected from the intertidal zone of the Yangtze Estuary were investigated using live observation, protargol staining and SSU rDNA sequencing. Both new species possess a unique S-shaped synhymenium that encircles the body, and differ from other congeners in body size, shape, and the pattern of contractile vacuoles. Phylogenetic tree results indicate that *Zosterodasys* may not be a monophyletic group. This research not only provides more detailed descriptions and genetic information of the previously poorly understood group of ciliates but also enriches our understanding of the protist diversity in the Yangtze Estuary. The finding provides fundamental data for future ecological monitoring and conservation efforts in such environments.

## 1. Introduction

Ciliated protists are a group of ubiquitous and diverse unicellular eukaryotes that play an important role in microbial food webs in both pelagic and benthic ecosystems [1,2,3]. They are also good indicators and have been employed for monitoring the quality of various aquatic habitats [4,5,6,7,8].

The class Phyllopharyngea Small and Lynn, 1981 is a specialized ciliate group. Basal bodies of its somatic kineties have a laterally directed kinetodesmal fibril and a reduced (or absent) transverse microtubular ribbon, and its cytostome possesses a pharyngeal tube which is surrounded by phyllae or leaf-like ribbons of microtubules [9]. This group exhibits high niche diversity and comprises free-living, commensal, and parasitic forms [10,11,12,13,14,15]. Recently, many new taxa within the class Phyllopharyngea were reported, indicating its diversity is underestimated. However, most studies focus on the subclass Cyrtophoria Fauré-Fremiet in Corliss, 1956, the most speciose group within Phyllopharyngea, and other groups, e.g., Synhymeniida de Puytorac et al., 1974, remain rarely investigated.

The affiliation of Synhymeniida was ambiguous for a long time. It used to be regarded as a member of the subclass Hypostomata Schewiakoff, 1896 or the class Nassophorea Small and Lynn, 1981 [9,16,17]. However, a recent study based on both the SSU rDNA phylogeny and the ultrastructure of kinetosomes and cytostomes suggested that Synhymeniida should represent a subclass within Phyllopharyngea [18]. This conclusion was supported by later molecular phylogenetic analyses based on multiple loci or both genomic and transcriptomic data [18,19,20,21,22]. However, only 16 SSU rDNA sequences, one LSU rDNA sequence, and one alpha-tubulin gene sequence are currently available, and the phylogeny of most members remains unresolved due to limited molecular data.

*Zosterodasys* Deroux, 1978 is one of four typical genera (*Zosterodasys*, *Nassulopsis* Foissner, Berger, & Kohmann, 1994, *Chilodontopsis* Blochmann, 1895 and *Orthodonella* Bhatia, 1936) within the subclass Synhymenia de Puytorac et al. in Deroux, 1978. It was originally described by Deroux (1978) and identified by (i) an obovoidal-to-ellipsoidal cell body lacking anterior rostral differentiation; (ii) a prominent cyrtos (an obconical cytopharyngeal apparatus reinforced by nematodesmal rods); and (iii) a distinctive ciliary structure termed the synhymenium, which extends obliquely from the left dorsal to the right ventral cell surface [22,23]. Vďačný and Tirjaková (2012) reviewed this genus in detail, and found among 31 nominal species only nine were reliable due to extensive synonyms and overly superficial original descriptions, indicating that further investigations are required [23].

Our recent investigations revealed that the Yangtze Estuary harbors high ciliate diversity [24,25,26,27,28,29]. As a new contribution, we isolated two *Zosterodasys* species in this region and found both of them represent previously undescribed species after detailed morphological investigations.

## 2. Materials and Methods

### 2.1. Sample Collection, Observations, and Identification

**Figure 1:**
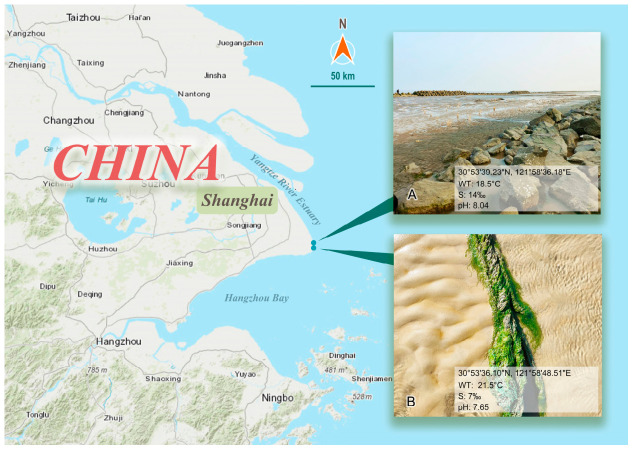
Locations and images of sampling sites. (**A**) Sampling site where the type population of *Zosterodasys paraminutus* nov. sp. was collected. (**B**) Sampling site where the type population of *Zosterodasys shanghaiensis* nov. sp. was collected.

Both species were collected from the intertidal zone in Nanhui Wetland, Shanghai, China. *Zosterodasys paraminutus* sp. nov. was collected from a tide pool (30°53′39.23″ N, 121°58′36.18″ E) on 12 March 2024 with water temperature 18.5 °C, pH 8.04 and salinity 14‰ (Figure 1A). A sponge and a toothbrush were used to gently scrub the surface biofilm and algae from the tide pool. The dislodged material, along with the surrounding seawater, was then drawn up using a pipette for collection. *Zosterodasys shanghaiensis* sp. nov. was collected from the sediment (30°53′36.10″ N, 121°58′48.51″ E), covered by a fishing net and *Enteromorpha* sp. on 18 April 2024 with water temperature 21.5 °C, pH 7.65 and salinity 7‰ (Figure 1B). A small pointed shovel was used to collect the surface sediment and seawater from the sampling site, together with the overlying cover (fishing net and *Enteromorpha* sp.), and all were placed into sampling containers.

The morphological study was immediately conducted after the samples were transported to the laboratory. Attempts to establish clonal cultures failed, and both *Zosterodasys* species were only maintained for approximately one week in the raw culture. Live cells were isolated using micropipettes and observed with a bright field and differential interference contrast microscope (Olympus BX53, Tokyo, Japan). Wilbert’s protargol method [30] was employed to reveal the ciliary pattern with a house-made protargol reagent [31]. All measurements and counts of live cells and stained specimens were performed under magnifications of 100× to 1000×. Drawings of live and protargol-stained cells were generated based on microphotographs. Taxonomic terminology was according to [9,23], and systematics mainly followed [18,20].

### 2.2. DNA Extraction, PCR Amplification, and Sequencing

For each species, a single cell was isolated and washed at least five times with sterile habitat water, and then starved for approximately 6 h until the cytoplasm became transparent and no algal cells were visible to minimize the risk of sample contamination. Genomic DNA was extracted using the DNeasy Blood and Tissue Kit (Qiagen, Hilden, Germany) following the manufacturer’s instructions. SSU rRNA gene was amplified by PCR using the universal eukaryotic primers 18SF (5′-AAC CTG GTT GAT CCT GCC AGT-3′) and 5.8 SR (5′-TAC TGA TAT GCT TAA GTT CAG CGG-3′) [32,33]. The protocols of PCR amplification followed [26,34]. The PCR products of single-cell samples were sent for Sanger sequencing. Sequence assembly and determination of SSU rRNA gene sequence followed [34].

### 2.3. Phylogenetic Analyses

Except for the two new sequences of *Zosterodasys paraminutus* sp. nov. and *Z. shanghaiensis* sp. nov., a set of 21 sequences were retrieved from the GenBank database and employed for phylogenetic analysis. *Ephelota gemmipara* (EU600180), *Hypocoma acinetarum* (JN867019), *Chlamydodon caudatus* (JQ904058), and *Trithigmostoma steini* (X71134) were designated as the outgroup. The SSU rRNA gene sequences were aligned and trimmed using the GUIDANCE2 web-server (https://guidance.tau.ac.il/, accessed on 16 June 2026) [35,36,37].

The best-fit substitution model (GTR + I + G) was determined using jModelTest2 ver. 2.1.6 on the CIPRES Science Gateway (https://www.phylo.org/, accessed on 16 June 2026) [38]. Phylogenies were inferred using both maximum likelihood (ML) and Bayesian inference (BI) methods on CIPRES. ML analysis with 10,000 ultrafast bootstrap replicates was performed in RAxML-HPC2 on ACCESS ver. 8.2.12. BI analysis was conducted using MrBayes ver. 3.2.7a, with four independent runs, each with four chains (nruns = 4), each with four Markov chains (nchains = 4). The analysis ran for 1,000,000 generations, sampling every 100 generations. Convergence among runs was assessed every 5000 generations using the average standard deviation of split frequencies, and analyses were considered converged when this value fell below 0.01. The first 25% of sampled trees were discarded as burn-in, and the remaining trees were used to generate a 50% majority-rule consensus tree. Posterior probabilities were calculated from the post-burn-in samples [39]. All resulting tree topologies were visualized in FigTree v1.4.3 (https://tree.bio.ed.ac.uk/software/figtree/, accessed on 16 June 2026).

The objective was to test the following hypotheses: (1) all *Zosterodasys* forms a monophyletic group; (2) all *Zosterodasys* except *Zosterodasys* sp.5 (KX302702) are monophyletic; (3) all *Zosterodasys* except *Zosterodasys* sp.6 (MZ098634) are monophyletic. To do this, an approximately unbiased (AU) test was conducted (Table S1) [40]. Constrained ML trees were generated using the same toolkit as for the unconstrained ML tree on the CIPRES Science Gateway, with specific topological constraints applied [41]. Site-specific likelihood values for both constrained and unconstrained ML topologies were calculated with a local installation of RAxML version 8.2.10, employing a partitioned GTRGAMMA model. The resulting likelihood scores were then processed using CONSEL version 0.1i under default settings to obtain *p*-values [42,43].

## 3. Results

### 3.1. Morphology and Taxonomy

Order Synhymeniida de Puytorac et al. in Deroux, 1978.

Family Orthodonellidae Jankowski, 1968.

Genus *Zosterodasys* Deroux, 1978.

**Figure 2:**
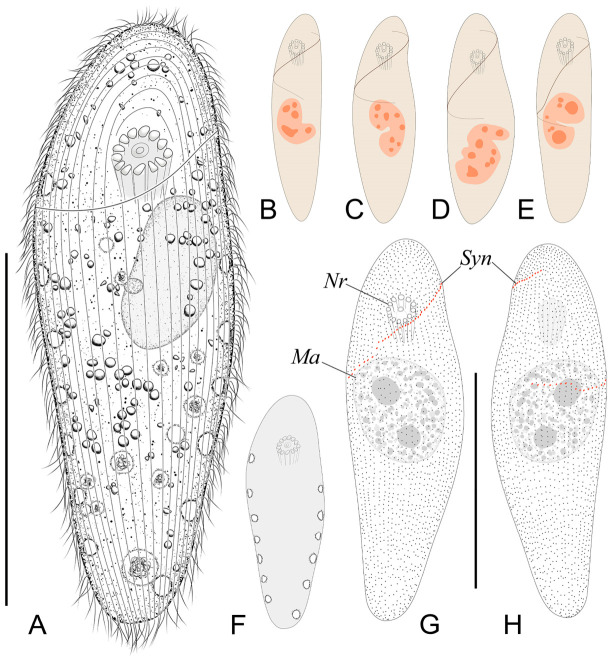
Morphology of *Zosterodasys paraminutus* nov. sp. from life (**A**,**F**) and after protargol staining (**B**–**E**,**G**,**H**). (**A**) Ventral view of a representative individual. (**B**–**E**) Showing the macronucleus with variable shapes. (**F**) Distribution pattern of contractile vacuoles. (**G**,**H**) Ventral (**G**) and dorsal view (**H**) of the holotype. Ma, macronucleus; Nr, nematodesmal rods; Syn, synhymenium. Scale bars: 60 μm.

**Figure 3:**
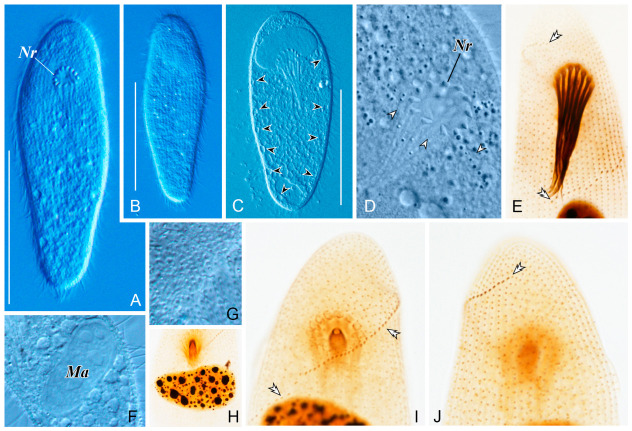
Morphology of *Zosterodasys paraminutus* nov. sp. from life (**A**–**D**,**F**,**G**) and after protargol staining (**E**,**H**–**J**). (**A**,**B**) ventral view (**A**) and dorsal view (**B**) of typical individual. (**C**) Arrowheads point to the contractile vacuoles. (**D**) Oral structure, arrowheads showing the synhymenium. (**E**) Double arrows point to the terminal of synhymenium on the dorsal side. (**F**,**H**) Macronucleus. (**G**) Detail of cell, showing the alveolar layer on the surface. (**I**,**J**) Ciliature in the anterior portion of ventral side (**I**) and dorsal side (**J**), double arrows point the terminal of synhymenium. Ma, macronucleus; Nr, nematodesmal rods. Scale bars: 60 μm.

1. *Zosterodasys paraminutus* sp. nov. (Figure 2A–H and Figure 3A–J; Table 1)

ZooBank registration number of the new species: urn:lsid:zoobank.org:act:149304E0-A90F-4422-A45A-F95FFA303E7E.

Diagnosis: Body size in vivo about 87–130 × 28–50 μm; elongated oval in outline; single macronucleus and one micronucleus; 39–59 somatic kineties; 12–15 nematodesmal rods; contractile vacuoles arranged along right and left margins of cell; synhymenium completely encircles cell; brackish water habitat.

Type locality: Intertidal zone in Nanhui Wetland, Shanghai, China (30°53′39.23″ N, 121°58′36.18″ E). Brackish.

Type material: A protargol slide (registration no. GJQ2024031202-1) with the holotype circled with black ink and two paratype slides (GJQ2024031202-2, GJQ2024031202-3) have been deposited in the Laboratory of Eukaryotic Microbiology, Shanghai Ocean University.

Etymology: The species-group name “*paraminutus*” is a composite of the Greek adjective “*para-*” (beside) and the species-group name “*minutus*”, indicating that it closely resembles *Zosterodasys minutus* Gong et al., 2007.

Morphological description: Body size about 87–130 × 28–50 μm in vivo, and about 80–123 × 22–38 μm after protargol staining. Cell slightly soft. Body narrowly obovate in outline with anterior and posterior ends broadly round, and the anterior end slight wider than posterior end. Both margins convex with anterior portion slightly projected to left (Figure 2A and Figure 3A,B). Cytostome located at anterior 1/5 of cell length (Figure 2A and Figure 3A,D), surrounded by 12–15 nematodesmal rods (14–39 μm after protargol staining). Cytoplasm colorless, containing lots of granules (1–2 μm across) and several food vacuoles (3–4 μm across). Macronucleus ovoid, about 25 × 10 μm in vivo, usually centrally located, and occasionally observed in the anterior portion of the cell, even in front of the cytostome (Figure 2A and Figure 3B,F). After protargol staining, the macronucleus exhibits a high diversity of shapes (Figure 2B–E and Figure 3H) from oval to irregular shape. Single micronucleus only observed in vivo, close to the macronucleus, about 2 μm in diameter. About 10–16 contractile vacuoles arranged in two rows, one each along left and right margins of cell; each contractile vacuole about 3–4 μm in diameter, pulsating every 19 s (Figure 2F and Figure 3C). Cilia about 6–7 µm long in vivo, uniformly distributed on ventral and dorsal surfaces. Movement moderately fast, sliding on the sediment surface.

Synhymenium completely encircles cell, composed of 40–73 loosely spaced dikinetids, extending from the anterior 1/12 of the dorsal side, curving around the ventral side, and running obliquely to about 1/2 of the dorsal side (Figure 2B–E,G,H and Figure 3E,I,J). In total 39–59 somatic kineties, and about 16–23 ventral kineties curving around and anterior to cytostome (Figure 2G and Figure 3I).

SSU rDNA sequence: The SSU rDNA sequence of *Zosterodasys paraminutus* sp. nov. (GenBank accession no. PZ466715) is 1704 bp in length with a GC content of 45.19%. It differs from its congeners by 22–87 nucleotides, with sequence identities ranging from 91.6% to 98.6% (Table 2).

**Figure 4:**
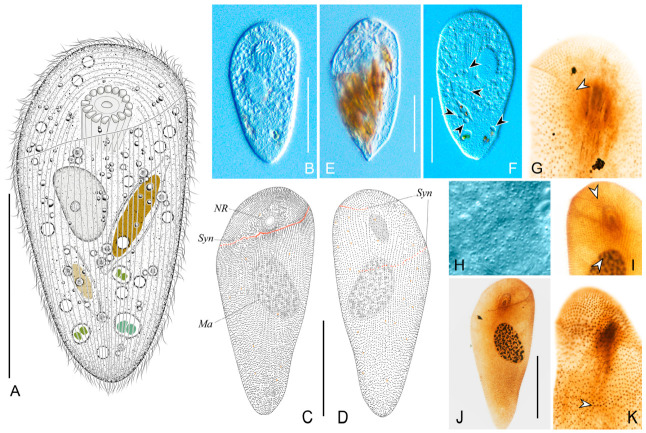
Morphology of *Zosterodasys shanghaiensis* nov. sp. from life (**A**,**B**,**E**,**F**,**H**) and after protargol staining (**C**,**D**,**G**,**I**–**K**). (**A**,**B**) Ventral view of a representative individual. (**C**,**D**,**J**) Ventral (**C**,**J**) and dorsal view (**D**) of the holotype. (**E**) An individual with diatoms in its cytoplasm. (**F**) Distribution pattern of contractile vacuoles, arrowheads point to contractile vacuoles. (**G**,**I**,**K**) Anterior portion of dorsal view, arrowheads indicate the terminal of synhymenium. (**H**) Cortical granules on the surface. Ma, macronucleus; Nr, nematodesmal rods; Syn, synhymenium. Scale bars: 60 μm (**A**,**B**–**G**), 80 μm (**C**,**D**,**J**).

2. *Zosterodasys shanghaiensis* sp. nov. (Figure 4A–K; Table 1)

ZooBank registration number of the new species: urn:lsid:zoobank.org:act:E1CF573D-F2F0-4AA7-B278-018171269971.

Diagnosis: Body size in vivo about 114–174 × 50–85 μm; obovate in outline; cortical granules distinct and arranged in rows between the kineties; single ellipsoidal macronucleus and one micronucleus; 74–122 somatic kineties; 12–17 nematodesmal rods; contractile vacuoles scattered in cytoplasm; synhymenium completely encircles cell; brackish water habitat.

Type locality: Intertidal zone on Cape Nanhui Sea-viewing Park, Shanghai, China (30°53′36.10″ N, 121°58′48.51″ E). Brackish.

Type material: A protargol slide (registration no. GJQ2024041801-1) with the holotype circled with black ink and two paratype slides (GJQ2024041801-2, GJQ2024041801-3) have been deposited in the Laboratory of Protozoology, Ocean University of China.

Etymology: The species-group name “*shanghaiensis*” indicates the place (Shanghai) where the species was originally discovered.

Morphological description: Body size about 114–174 × 50–85 μm in vivo, and about 88–197 × 30–87 μm after protargol staining. Cell inflexible. Body obovate in outline with anterior end broadly rounded and posterior end tapered. Right margins convex with anterior portion slightly projected to left (Figure 4A,B,E). Cyrtos surrounded by 12–17 nematodesmal rods (Figure 4A,B), about 19–46 μm long after protargol staining (Figure 4C,J). Cytostome located at anterior 1/5 of cell length. Densely spaced cortical granules underneath the cell surface, arranged in rows between somatic kineties, each about 0.5–1 μm in diameter (Figure 4F). Cytoplasm colorless with numerous granules about 1–2 μm. Some individuals containing numerous undigested diatoms and green spherical algae, rendering cell yellowish–green (Figure 4E). Macronucleus ovoid located in the middle of cell, size about 35 × 19 μm in vivo, 29–58 × 10–35 μm in stained specimens (Figure 4A,C,J). One globule micronucleus close to the macronucleus about 2–3 μm in diameter. Numerous contractile vacuoles scattered irregularly throughout the cytoplasm, diameter about 3–4 μm, that contract every 25–30 s (Figure 4F). Cilia about 5–6 µm long in vivo, densely and uniformly implanted on ventral and dorsal surfaces.

Synhymenium completely encircles cell, composed of 70–135 densely spaced dikinetids, extending from the anterior 1/10 of the dorsal side, curving around the ventral side, and running obliquely to approximately 1/3 of the dorsal side (Figure 4C,D,G,I,K). In total 74–122 somatic kineties, and about 36–41 ventral kineties curving around and anterior to cytostome.

SSU rDNA sequence: The SSU rDNA sequence of *Zosterodasys shanghaiensis* sp. nov. (GenBank accession no. PZ466716) is 1705 bp in length with a GC content of 45.10%. It differs from its congeners by 1–86 nucleotides, with sequence identities ranging from 92.9% to 99.5% (Table 2).

### 3.2. Phylogenetic Analyses

**Figure 5:**
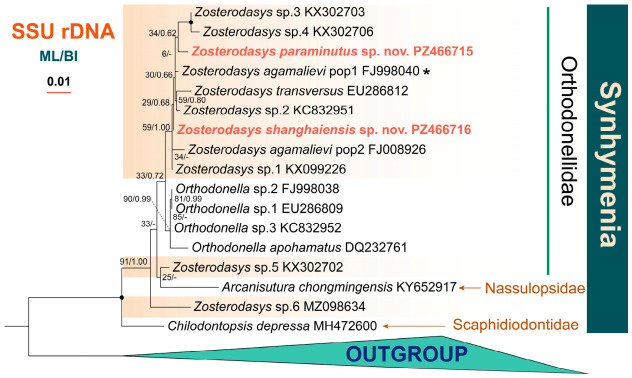
Maximum likelihood (ML) tree inferred from SSU rRNA gene sequences, showing the phylogenetic positions of *Zosterodasys paraminutus* sp. nov., *Zosterodasys shanghaiensis* sp. nov. (in tangerine). Numbers at nodes show ML bootstrap values and BI posterior probabilities (ML/BI). Black dots indicate fully supported nodes (100% ML/1.00 BI). “-“ indicates the disagreement between ML and BI trees. The scale bar corresponds to 1 substitution per 100 nucleotide positions. “*” indicates that the sequence is named *Chilodontopsis* sp. in NCBI, but is named *Zosterodasys agamalievi* in [21,44,45].

The topologies of the ML and BI trees were largely congruent. Therefore, only the ML tree is presented, displaying both bootstrap values and posterior probabilities. The newly sequenced species clustered with most of its congeners. However, after adding these new sequences, the genus *Zosterodasys* did not appear monophyletic: *Zosterodasys* sp.5 (KX302702) and *Zosterodasys* sp.6 (MZ098643) diverged from the remaining members of the genus and fell outside the family Orthodonellidae. Specifically, *Zosterodasys* sp.5 (KX302702) clustered with *Arcanisutura chongmingensis* (KY652917) with very low support (25% ML), while *Zosterodasys* sp.6 branched to the clade comprising Orthodonellidae and Nassulopsidae with high support (91% ML, 1.00 BI). Both new species were nested within the family Orthodonellidae and grouped with other *Zosterodasys*. *Zosterodasys paraminutus* sp. nov. was grouped with *Zosterodasys* sp.3 (KX302703) and *Zosterodasys* sp.4 (KX302706) with low support (34% ML, 0.62 BI), and then formed a clade with *Z. agamalievi* pop1 (FJ998040), *Z. transversus* (EU286812) and *Zosterodasys* sp.2 (KC832951), which was sister to *Z. shanghaiensis* sp. nov. with low support (29% ML, 0.68 BI) (Figure 5).

## 4. Discussion

### 4.1. Comparison of Zosterodasys paraminutus sp. nov. with Congeners

A previous study reviewed the genus *Zosterdasys* Deroux, 1978 and considered characteristics of the synhymenium, body size, number of ciliary rows, number and arrangement of contractile vacuoles, and number of nematodesmal rods as important characteristics for species identification within this genus [23].

To date, nine *Zosterodasys* species are valid and five are classified as species inquirendae [23]. Among them, only *Z. minutus* and *Z. henarensis* Fernandez-Leborans & Alekperov, 1996 possess a synhymenium completely encircling the cell. According to the ciliary pattern, *Z. paraminutus* exhibits the greatest similarity to *Z. minutus*. They share a similar body size in vivo (87–130 × 28–50 μm in *Z. paraminutus* sp. nov. vs. 50–100 × 20–40 μm in *Z. minutus*), number of nematodesmata rods (11–13 in *Z. paraminutus* sp. nov. vs. 10–12 in *Z. minutus*), number of somatic kineties (39–59 in *Z. paraminutus* sp. nov. vs. 34–55 in *Z. minutus*) and a synhymenium that completely encircles the body, which is composed of loosely distributed dikinetids. However, *Z. paraminutus* sp. nov. can be distinguished from *Z. minutus* by the shape of the posterior end (rounded vs. tapered) and the distribution pattern of contractile vacuoles (arranged along the left and right margin of the cell vs. irregularly distributed). Furthermore, *Z. minutus* possesses a distinct meridional suture in the posterior half of the cell, which is absent in *Z. paraminutus* sp. nov. [46].

In addition, unlike *Zosterodasys henarensis*, *Z. paraminutus* sp. nov. possesses an elongated oval body shape (vs. cylindrical shape with a constriction in the anterior quarter), 39–59 somatic kineties (vs. 70–80), and 11–13 nematodesmal rods (vs. 17–20) [47].

Although the structure of the synhymenium in *Zosterodasys caudatus* and *Z. numerosus* remains unclear, *Z. caudatus* can still be distinguished from *Z. paraminutus* sp. nov. by a shorter body length in vivo (60–80 μm vs. 87–130 μm), the number of contractile vacuoles (one vs. many), body shape (narrowly obovate with tail-like posterior end curved rightwards vs. elongated oval body shape with both ends round), and the habitat (marine vs. brackish water). *Zosterodasys numerosus* exhibits a larger body in vivo (200–250 × 55–70 μm vs. 87–130 × 28–50 μm) and different habitat (marine habitat vs. brackish water habitat) [48,49].

### 4.2. Comparison of Zosterodasys shanghaiensis sp. nov. with Congeners

Among those *Zosterodasys* species with a synhymenium encircling the cell, *Z. shanghaiensis* sp. nov. differs from *Z. henarensis* by body shape (obovate vs. slender elongated oval) and the number of nematodesmal rods (12–17 vs. 17–20). Compared to *Z. minutus*, *Z. shanghaiensis* sp. nov. possesses more nematodesmal rods (12–17 vs. 10–12) and its ventral somatic kineties do not form a suture (vs. a suture present in the meridional and posterior half of the cell) [46,47].

Unlike the other new species, *Zosterodasys paraminutus* sp. nov., *Z. shanghaiensis* nov. sp. possesses 74–122 somatic kineties (vs. 39–59), 12–17 nematodesmal rods (vs. 11–13), and irregularly distributed contractile vacuoles (vs. contractile vacuoles arranged regularly along the left and right margins). Furthermore, their body shape is different as well, i.e., *Z. shanghaiensis* sp. nov. has an elongated oval shape with both ends round while *Z. paraminutus* sp. nov. has an obovate shape with the anterior end broadly round and the posterior end tapered.

Furthermore, *Zosterodasys shanghaiensis* sp. nov. resembles *Z. agamalievi* and *Z. transversus* in size in vivo (114–174 × 50–85 μm in *Z. shanghaiensis* sp. nov. vs. 90–150 × 30–55 μm in *Z. agamalievi*, 120–240 × 50–115 μm in *Z. transversus*), in somatic kinety number (74–122 in *Z. shanghaiensis* sp. nov. vs. 50–95 in *Z. agamalievi*, and approximately 82 in *Z. transversus*), and in nematodesmata rods number (12–17 in *Z. shanghaiensis* sp. nov. vs. 10–16 in *Z. agamalievi* vs. 12–16 in *Z. transversus*). However, *Z. shanghaiensis* sp. nov. is clearly distinguished from both by the pattern of the synhymenium (completely encircling the body in *Z. shanghaiensis* sp. nov. vs. incompletely encircling in the latter two). Moreover, according to hand drawings from the original description of *Z. agamalievi*, it possesses only two contractile vacuole pores, whereas *Z. shanghaiensis* sp. nov. has 4–27. Additionally, *Z. transversus* is found exclusively in freshwater, while *Z. shanghaiensis* sp. nov. inhabits brackish water [14,22,23].

### 4.3. Phylogenetic Analyses

With the addition of two new sequences, the Synhymenia were still monophyletic, but Orthodonellidae and *Zosterodasys* were not, as *Zosterodasys* sp.5 (KX302702) and *Zosterodasys* sp.6 (MZ098634) diverged from its congeners and was placed outside the family Orthodonellidae. The result of the AU test rejected the monophyly of the genus *Zosterodasys* (*p*-value = 9 × 10^−41^ < 0.05). Moreover, the monophylies of either all *Zosterodasys* except *Zosterodasys* sp.5 (KX302702) (*p*-value = 0.004 < 0.05) or all *Zosterodasys* except *Zosterodasys* sp.6 (MZ098634) (*p*-value = 1 × 10^−40^ < 0.05) were rejected (Table S1). Discrimination of genera within the Synhymenia was mainly based on the details of the ciliature. Given the lack of corresponding morphological information for *Zosterodasys* sp.5 (KX302702) and *Zosterodasys* sp.6 (MZ098634) and their divergent positions from the core of *Zosterodasys* in the phylogenetic trees, these two sequences may have been misidentified as *Zosterodasys*.

In the results of phylogenetic analyses, the two newly sequenced species clustered with the majority of other *Zosterodasys* species, including the type species *Z. agamalievi*. This undoubtedly provides further support for the assignment of these two new species to the genus *Zosterodasys*. The SSU rDNA sequence of *Z. shanghaiensis* sp. nov. differs from that of *Zosterodasys* sp.1 (KX099226) by only one nucleotide, which strongly suggests that they may represent the same species (Table 2). However, because *Zosterodasys* sp.1 (KX099226) lacks corresponding morphological information, its SSU rDNA sequence is incomplete (only 1586 bp), and the two sequences do not form a clade in the phylogenetic tree, we refrain from drawing a hasty conclusion to avoid further confusion.

It is noteworthy that two sequences under the species name *Zosterodasys agamalievi* fell in different clades, indicating a misidentification rather than high intragenomic rDNA polymorphism [21]. Although both sequences were submitted by protozoologists, neither of them was reported with detailed morphological features. More caution should be taken when identifying *Zosterodasys* species. Additionally, a recent study restored the relationships among the other four subclasses of Phyllopharyngea using structural and ontogenetic bodies of evidence [50]. Therefore, obtaining further structural and ontogenetic information on newly included species within Synhymenia is of great importance for clarifying the phylogenetic relationships among various groups within Phyllopharyngea.

### 4.4. Key to the Identification of Zosterodasys Species Update

1. The synhymenium completely encircles the body..............................................................2The synhymenium incompletely encircles the body...............................................................52. Number of somatic kineties ≥ 70............................................................................................3Number of somatic kineties < 70................................................................................................43. Body constricted in the anterior quarter............................................................*Z. henarensis*No distinct body constriction..............................................................*Z. shanghaiensis* sp. nov.4. Contractile vacuoles arranged along both the left and right margins of the cell............................................................................................................................................................................................................................................................*Z. paraminutus* sp. nov.Contractile vacuoles scattered throughout the body...............................................*Z. minutus*5. The posterior body end is tail-like........................................................................*Z. caudatus*The posterior body end is tapering, narrowly or broadly rounded......................................66. One single contractile vacuole....................................................................................*Z. debilis*Multiple contractile vacuoles......................................................................................................77. Contractile vacuoles constricted in specific regions of the body .....................................8Contractile vacuoles scattered throughout the body..............................................................118. Contractile vacuoles only positioned in the posterior portion of the body....*Z. hisioensis*Contractile vacuoles only positioned along the cell margins.................................................99. Three contractile vacuoles arranged along the right margin................................*Z. acutus*Six to nine contractile vacuoles evenly arranged along both the left and right cell margins...............................................................................................................................................1010. Body length 50–60 μm...............................................................................................*Z. minor*Body length > 100 μm...............................................................................................*Z. kryophilus*11. The posterior end is tapered or pointed............................................................*Z. numerous*The posterior end is rounded....................................................................................................1212. Post-synhymenial ventral kineties form a suture in the posterior body half..*Z. derouxi*Post-synhymenial ventral kineties do not form a suture.......................................................1313. Size about 90–150 × 30–55 μm in vivo. Marine habitats.................................*Z. agamalievi*Size about 120–240 × 50–115 μm in vivo. Freshwater habitats..........................*Z. transversus*

## 5. Conclusions

Both morphological and molecular data support the establishment of two new species, *Zosterodasys paraminutus* sp. nov. and *Z. shanghaiensis* sp. nov. And the key for the species within *Zosterodasys* was updated, which will facilitate identification. Although two new sequences were added, molecular information within this genus is still extremely limited and almost all available sequences lack corresponding morphological data. Therefore, future efforts should cover molecular information from more species to resolve the phylogeny of the genus *Zosterodasys*.

## Figures and Tables

**Table 1:** Morphometric characteristics of *Zosterodasys paraminutus* sp. nov. (upper) and *Zosterodasys shanghaiensis* sp. nov. (lower) based on protargol stained specimens.

Characters	Min	Max	Mean	M	SD	CV %	N
Body length	80	123	103.6	102.5	12.5	12.1	20
	89	198	138.2	133.0	25.7	18.5	17
Body width	22	38	29.9	30.5	4.6	15.4	20
	30	87	53.6	53.0	11.5	21.4	18
Length of Ma	20	38	30.1	30.0	4.7	15.6	20
	29	58	42.7	41.0	7.7	18.2	18
Width of Ma	10	29	16.8	15.5	3.7	21.9	20
	10	35	21.8	20.0	6.5	30.1	18
No. of pre-synhymenium SK	16	23	20.0	21.0	2.4	11.9	19
	26	41	33.9	34.0	4.2	12.5	17
No. of post-synhymenium SK	34	55	46.7	48.0	5.5	11.9	19
	62	109	86.1	90.0	14.0	16.2	17
No. of SK	39	59	46.9	47.0	4.6	9.8	19
	74	122	96.5	96.0	10.2	10.6	18
No. of SK covered by synhymenium	34	55	45.3	45.0	5.2	11.4	19
	62	109	81.4	78.0	12.5	15.4	17
No. of dikinetids in synhymenium	40	73	57.0	58.0	7.6	13.2	20
	70	135	103.9	104.0	14.4	13.8	18
No. of nematodesmata rods	11	13	12.2	12.0	0.6	4.7	20
	12	17	14.5	15.0	1.5	10.0	17
Diameter of cytostome.	2	5	3.4	3.0	0.9	27.6	20
	3	8	5.4	5.0	1.2	21.6	18
Length of cyrtos	14	39	23.5	22.0	6.0	25.7	20
	19	46	30.9	32.0	6.7	21.7	18
No. of CVP	–	–	–	–	–	–	–
	4	27	11.6	11.0	6.3	54.7	18

All measurements in µm. Abbreviations: CV, coefficient of variation in %; CVP, contractile vacuole pores; M, median; Ma, macronucleus; Max, maximum; Min, minimum; N, number of specimens measured; SD, standard deviation; SK, somatic kinety; –, data not available.

**Table 2:** Sequence identity (above diagonal) and sequence difference count (below diagonal) between the two newly sequenced *Zosterodasys* species and their congeners based on SSU rDNA sequences.

Species	GenBank Number	Length	1	2	3	4	5	6	7	8	9	10	11
1. *Z. paraminutus* sp. nov.	PZ466715	1704	-	0.987	0.982	0.987	0.984	0.939	0.985	0.941	0.941	0.939	0.961
2. *Z. shanghaiensis* sp. nov.	PZ466716	1705	22		0.993	0.995	0.987	0.999	0.997	0.941	0.941	0.939	0.961
3. *Z. transversus*	EU286812	1746	31	12		0.989	0.981	0.992	0.993	0.933	0.933	0.929	0.955
4. *Z. agamalievi* pop 1	FJ998040	1749	22	8	19		0.983	0.994	0.993	0.941	0.941	0.939	0.959
5. *Z. agamalievi* pop 2	FJ008926	1749	27	22	34	30		0.989	0.984	0.941	0.941	0.939	0.964
6. *Zosterodasys* sp.1	KX099226	1586	23	1	13	9	17		0.996	0.941	0.941	0.939	0.960
7. *Zosterodasys* sp.2	KC832951	1752	25	6	13	12	29	7		0.936	0.936	0.934	0.959
8. *Zosterodasys* sp.3	KX302703	1387	80	80	85	81	81	64	83		0.996	0.941	0.937
9. *Zosterodasys* sp.4	KX302706	1383	86	85	90	86	86	88	20	5		0.986	0.937
10. *Zosterodasys* sp.5	KX302702	1386	87	86	92	87	87	67	89	16	20		0.936
11. *Zosterodasys* sp.6	MZ098634	1709	66	67	77	70	62	64	71	94	99	95	

## Data Availability

The original data presented in the study are openly available in the National Center for Biotechnology Information https://www.ncbi.nlm.nih.gov/ (accessed on 29 May 2026).

## Supplementary Materials

The following supporting information can be downloaded at: https://www.mdpi.com/article/10.3390/ani16121930/s1, Table S1: Approximately unbiased (AU) test results based on SSU rDNA sequences. Table S2: Morphometric comparison of *Zosterodasys paraminutus* sp. nov. and *Z. shanghaiensis* sp. nov. with its congeners. References [51,52,53,54,55] are cited in the Supplementary Materials.

## Abbreviations

The following abbreviations are used in this manuscript:

BI Tree	Bayesian Inference Tree
CV	Coefficient of Variation
CVP	Contractile Vacuole Pore
M	Median
Ma	Macronucleus
Max	Maximum
Min	Minimum
ML Tree	Maximum Likelihood Tree
N	Number of Specimens Measured
Nr	Nematodesmal Rod
SD	Standard Deviation
SK	Somatic Kinety
SSU rDNA	Small Subunit Ribosomal DNA
Syn	Synhymenium
