# Supplementary material for: Taxonomy and Phylogeny of Two New Zosterodasys Species (Protista: Ciliophora: Phyllopharyngea) from the Yangtze Estuary, China
Source: Animals (Basel). 2026 Jun 22;16(12):1930. doi: 10.3390/ani16121930 (PMC13296273; doi:10.3390/ani16121930)
Supplement: Supplementary file 1 [file animals-16-01930-s001.zip › animals-4324825-supplementary.pdf]

Supplementary Table S1. Approximately unbiased (AU) tests results based on SSU rDNA sequences.

Hypothesis	*P*-value	Conclusion
All Zosterodasys forms a monophyletic group	9e-041	Rejected
All *Zosterodasys* except *Zosterodasys* sp.5 (KX302702) are monophyletic	0.004	Rejected
All Zosterodasys except *Zosterodasys* sp.6 (MZ098634) are monophyletic	1e-040	Rejected

Supplementary Table S2. Morphometric comparison of *Zosterodasys paraminutus* sp. nov. and *Z. shanghaiensis* sp. nov. with its congeners.

Species	Size in vivo (μm)	Body shape	No. of SK	No. of Nr	Structure of Synhymenium	No. of dikinetids in synhymenium	Pattern of CV	Habitat	Data source
Z. shanghaiensis sp. nov.	114–174 × 50–85	Obovate	74–122	12–17	Completely encircles body	70–135	Several, scattered	Brackish water	present work
Z. paraminutus sp. nov.	87–130 × 28–50	Elongated oval	39–59	11–13	Completely encircles body	44–73	Several, arranged along both sides of the cell	Brackish water	present work

Z. hisionensis	150–230 × 55–75	Obovate to elliptical	Ventral:45–47 Dorsal:30–36	16–22	Incompletely encircles body	87	One large posterior accompanied by 4–5 small ones	Marine	Ozaki and Yagiu, 1941
Z. transversus	120–240 × 50–115	Broadly to narrowly obovate or elliptical	Ventral: 36–52 Dorsal: 30–43	12–16	Incompletely encircles body	–	Several, scattered	Fresh water	Foissner et al., 1994
Z. agamalievi	90–150 × 30–55	Broadly to narrowly obovate or elliptical	50–95	10–16	Incompletely encircles body	–	Several, scattered	Marine	Deroux, 1978
Z. debilis	60–90 × 40–50	Elliptical	Ventral: 20–25 Dorsal: 10–13	10–13	Incompletely encircles body	–	1, occupying posterior body third	Fresh water	Vďačný and Tirjaková, 2012
Z. derouxi	250–300 × 90–125	Obovate to broadly obovate	68–100	12–15	Incompletely encircles body	–	3–5, scattered in posterior body portion	Fresh water	Aliyev, 1990
Z. kryophilus	100–170 × 50–60	Obovate to elliptical	Ventral: 25–36 Dorsal: 21–33	17–26	Incompletely encircles body	–	6–9, spaced along left postoral and posterior right body margin	Marine	Petz et al., 1995

Z. minor	50–60 × 30–40	Elliptical	Ventral: 18–21 Dorsal: 16–18	14–16	Incompletely encircles body	–	Several, spaced along left postoral and posterior right body margin	Marine	Alekperov et al., 2007
Z. minutus	50–100 × 20–40	Obovate	34–55	10–12	Completely encircles body	–	Several, scattered	Marine	Gong et al., 2007
Z. mirabilis	300–350 × 110–150	Obovate	Ventral: 30–60 Dorsal: 35–45	14–19	Incompletely encircles body	–	–	Fresh water	Vďačný and Tirjaková, 2012
Z. acutus	80–130 × 35–50	Narrowly obovate	Ventral: 22	16	–	–	3, arranged in a row near right margin in posterior body half	Marine	Ozaki and Yagiu, 1941
Z. caudatus	60–80	Narrowly obovate with tail-like posterior end	–	–	–	–	1, in mid-body on left ventral side	Marine	Vďačný and Tirjaková, 2012
Z. numerosus	200–250 × 55–70	Narrowly obovate	–	–	–	–	Several, scattered	Marine	Ozaki and Yagiu, 1941

Z. henarensis	165–265 × 40–70	Very narrowly elliptical with constriction in anterior body quarter	70–80	17–20	Completely encircles body	–	–	Fresh water	Fernandez-Leborans and Alekperov, 1996

* All measurements in. Abbreviations: CV, contractile vacuoles; Nr, nematodesmata rods; SK, somatic kinety; –, data not available.

Reference

Aliyev, A.R. Novye vidy infuzorij roda *Zosterodasys* (Synhymeniida) iz estestvennyh vodoemov Azerbajdzana. *Zool. Zh.* **1990**, 19, 13–23.

Deroux, G. The hypostome ciliate order Synhymeniida: from *Chilodontopsis* of Blochmann to *Nassulopsis* of Fauré-Fremiet. *Trans. Am. Microsc. Soc.* **1978**, 97(4), 458–469. https://doi.org/10.2307/3226163

Fernandez-Leborans, G.; Alekperov, I.K. Revision of the genus *Zosterodasys* with description of new species (Protozoa, Ciliophora). *Zoosyst. Ross.* **1996**, 4, 3–16.

Foissner, W.; Berger, H.; Kohmann, F. Taxonomische und Ökologische Revision der Ciliaten des Saprobiensystems – Band III: Hymenostomata, Prostomatida, Nassulida. Inf. *Ber. Bayer. Landesamtes Wasserwirtschaft* 1994, 548.

Gong, J.; Choi, J.K.; Roberts, D.M.; Kim, S.Y.; Min, G.S. Morphological descriptions of new and little-known benthic ciliates from Ganghwa tidal flat, Korea. *J. Eukaryot. Microbiol.* **2007**, 54, 306–316. https://doi.org/10.1111/j.1550-7408.2007.00268.x

Alekperov, I.; Edward, B.; Nataly, S. The free-living ciliates of the Mexican Gulf coast near Port Aransas city and its suburbs (South Texas, USA). *Protistology* **2007**, 5, 101–130.

Ozaki, Y.; Yagiu, R. Studies on the marine ciliates of Japan, mainly from the Setonaikai (the Inland Sea of Japan). *J. Sci. Hiroshima Univ., Ser. B, Div. 1* **1941**, 8, 165–184.

Petz, W.; Song, W.; Wilbert, N. Taxonomy and ecology of the ciliate fauna (Protozoa, Ciliophora) in the endopagial and pelagial of the Weddell Sea, Antarctica, ed. *Land Oberösterreich, OÖ Landesmuseum, Linz.* **1995**, 40, 1–223.

Vďačný, P.; Tirjaková, E. Taxonomic revision of the ciliate genus *Zosterodasys* Deroux, 1978 (Protista: Ciliophora: Synhymeniida). *Zootaxa* **2012**, 3345, 34–58. https://doi.org/10.11646/zootaxa.3345.1.2
